# Melanopsin-Expressing Amphioxus Photoreceptors Transduce Light via a Phospholipase C Signaling Cascade

**DOI:** 10.1371/journal.pone.0029813

**Published:** 2012-01-03

**Authors:** Juan Manuel Angueyra, Camila Pulido, Gerardo Malagón, Enrico Nasi, Maria del Pilar Gomez

**Affiliations:** 1 Marine Biological Laboratory, Woods Hole, Massachussets, United States of America; 2 Departamento de Biología, Universidad Nacional de Colombia, Bogotá, Colombia; 3 Instituto de Genética, Universidad Nacional de Colombia, Bogotá, Colombia; Dalhousie University, Canada

## Abstract

Melanopsin, the receptor molecule that underlies light sensitivity in mammalian ‘circadian’ receptors, is homologous to invertebrate rhodopsins and has been proposed to operate via a similar signaling pathway. Its downstream effectors, however, remain elusive. Melanopsin also expresses in two distinct light-sensitive cell types in the neural tube of amphioxus. This organism is the most basal extant chordate and can help outline the evolutionary history of different photoreceptor lineages and their transduction mechanisms; moreover, isolated amphioxus photoreceptors offer unique advantages, because they are unambiguously identifiable and amenable to single-cell physiological assays. In the present study whole-cell patch clamp recording, pharmacological manipulations, and immunodetection were utilized to investigate light transduction in amphioxus photoreceptors. A G_q_ was identified and selectively localized to the photosensitive microvillar membrane, while the pivotal role of phospholipase C was established pharmacologically. The photocurrent was profoundly depressed by IP_3_ receptor antagonists, highlighting the importance of IP_3_ receptors in light signaling. By contrast, surrogates of diacylglycerol (DAG), as well as poly-unsaturated fatty acids failed to activate a membrane conductance or to alter the light response. The results strengthen the notion that calcium released from the ER via IP_3_-sensitive channels may fulfill a key role in conveying - directly or indirectly - the melanopsin-initiated light signal to the photoconductance; moreover, they challenge the dogma that microvillar photoreceptors and phoshoinositide-based light transduction are a prerogative of invertebrate eyes.

## Introduction

A long-held belief, based on detailed morphological observations of eyes and photoreceptors across a wide variety of animal species, maintained that vision has evolved independently in different phyla [1]. In particular, microvillar photoreceptors, on one hand, and rods and cones on the other - the two canonical classes of light-sensing cells – have been thought to represent an instance of convergent evolution, and to be exclusively confined to invertebrates and vertebrates, respectively. Contrary to such view, recent lines of evidence derived from the molecular analysis of the genes that specify eye development and of the protein families that underlie light transduction, are gradually converging on the conclusion that visual mechanisms in metazoa may share a monophyletic origin [2]. In fact, microvillar photoreceptors are the likely descendants of the light sensor of the most primitive proto-eye already present in pre-bilateria [3], [4]; as such, one could expect to find them across both protostomia and deuterostomia. Representation of this ancient line of visual cells amongst the vertebrates had been traditionally dismissed, but has strongly re-emerged in recent times with the identification of unconventional photoreceptors in the ganglion layer of the mammalian retina [5], [6], dubbed *intrinsically photosensitive retinal ganglion cells* (*ipRGCs*). These cells subserve non-visual light-dependent functions, such as controlling the pupillary reflex and photo-entraining the circadian rhythms. Like invertebrate rhabdomeric visual cells, these ‘circadian’ photoreceptors depolarize to light, and their photopigment, melanopsin, has a significant similarity to the rhodopsin of invertebrates [7]; furthermore, clues have been garnered that the transduction cascade is PLC-dependent [8], [9]. Nonetheless, the characteristic microvillar cellular architecture is conspicuously absent in ipRGCs, and a detailed investigation of the effector mechanisms in native cells proved challenging, owing to their extreme scarcity and difficult identification. In the present report an alternative model system was utilized to address the nature of the light-signaling pathway that couples melanopsin photostimulation to the ion channels underlying the receptor potential. The approach adopted was to exploit an early chordate in which melanopsin-expressing cells are not as under-represented as in the retina of mammals, and have retained the distinguishing microvillar traits, making their classification unambiguous and facilitating their targeting in physiological studies. According to molecular phylogeny, amphioxus is the most basal living chordate [10]. Melanopsin has been detected in its neural tube [11], and its pattern of expression coincides with the distribution of two groups of cells, known as Joseph cells and organs of Hesse, respectively, which display distinct microvilli [12], [13], [14]. Our recent patch clamp measurements in enzymatically isolated cells of both types established that they are indeed primary photoreceptors, and their light response has an action spectrum similar to the absorption spectrum reported for amphioxus melanopsin *in vitro* [15]. The receptor potential of both Joseph and Hesse cells is depolarizing, and is accompanied by an increase in a cationic membrane conductance; furthermore, imaging with fluorescent indicators reveals that light mobilizes calcium from intracellular stores. In the present work, we utilized a multi-pronged approach to examine the nature of the enzymatic cascade that couples light absorption to ion channel activation. The significance of this endeavor is two-fold: on the one hand it can help elucidate the evolutionary history of microvillar photoreceptors and their representation amongst chordates; on the other hand, it provides clues on the light-signalling mechanisms of melanopsin in a native system.

## Results

**Figure 1:**
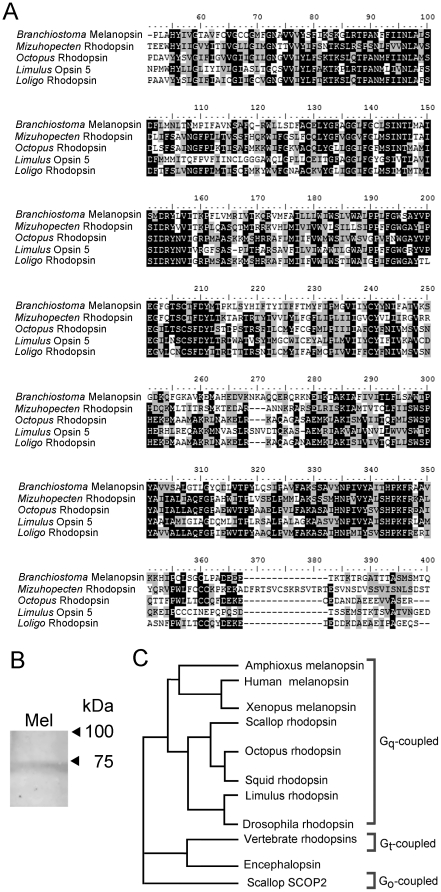
Amphioxus melanopsin groups with G_q_-coupled rhodopsins. (*A*) The translated sequence of *Branchiostoma* was subjected to a BLAST search, and subsequently aligned with the highest-ranking hits among *non*-melanopsin photopigments. All belong to microvillar photoreceptors from eyes of invertebrates. The Clustal W alignment shows the core region of the proteins (omitting the carboxy-terminus, and part of the amino-terminus, which are generally divergent); black shading indicates identity, whereas grey shading indicates conservative aminoacid substitutions. Accession numbers: amphioxus (*Branchiostoma*) Q4R1I4; scallop (*Mizuhopecten*), O15973.1, octopus (*Octopus*). P09241.1; horseshoe crab (*Limulus*), ACO05013.1; and squid (*Loligo*) P24603.1. (*B*) Western blot with anti-melanopsin antibodies. A single band of ≈71 kDa was detected, closely approaching the size expected from the predicted aminoacid sequence. This confirms that the melanopsin protein expresses in the neural tube of *B. floridae*. (*C*) Phylogenetic tree illustrating the grouping of the main classes of photopigments found in the animal kingdom. Amphioxus melanopsin is closely related to the G_q_-coupled rhodopsin of mollusks and arthropods.

The microvillar architecture of Joseph and Hesse cells of amphioxus is reminiscent of the rhabdomeric photoreceptors of invertebrates, and so are the functional properties of their photoresponse. Moreover, the predicted aminoacid sequence of amphioxus melanopsin resembles most closely, amongst non-melanopsin photopigments, the rhodopsin of microvillar visual cells of the scallop (*Mizuhopecten yessoensis*), octopus (*Octopus dofleini*), horseshoe crab (*Limulus polyphemus*), and squid (*Loligo forbesi*). In all cases the overall identity is 21–22%; by contrast, the similarity to the closest opsins from ciliary-type photoreceptors is substantially lower (15%–17% overall, far below all other reported microvillar photoreceptor rhodopsins). Figure 1A shows a multi-sequence alignment restricted to the core region of the amphioxus melanopsin (encompassing the 7 trans-membrane helices but excluding part of the N-terminus and the C-terminal region, where the greatest degree of divergence appears) against the aforementioned species, revealing a striking similarity. Panel B of the same figure shows a Western blot of amphioxus neural tube lysate, probed with antibodies raised against an extracellular domain of human melanopsin, that cross-react with other vertebrate forms (V-20, Santa Cruz Biotechnology). A single band was detected, with an apparent molecular weight of ≈71 kDa; this is somewhat higher than that of mammals (typically ≈49–62 kDa, depending on glycosylation state) [16], but compares favorably with the figure of ≈67 kDa from the predicted aminoacid sequence of *Branchiostoma floridae* melanopsin. Panel C of Figure 1 shows a simplified phylogenetic tree of representative animal photopigments, illustrating how amphioxus and vertebrate melanopsins group with the rhodopsins that are known to signal via G_q_. It is therefore plausible that the coupling of photon absorption to channel gating in amphioxus may also be based on the G_q_-triggered PLC signaling cascade.

**Figure 2:**
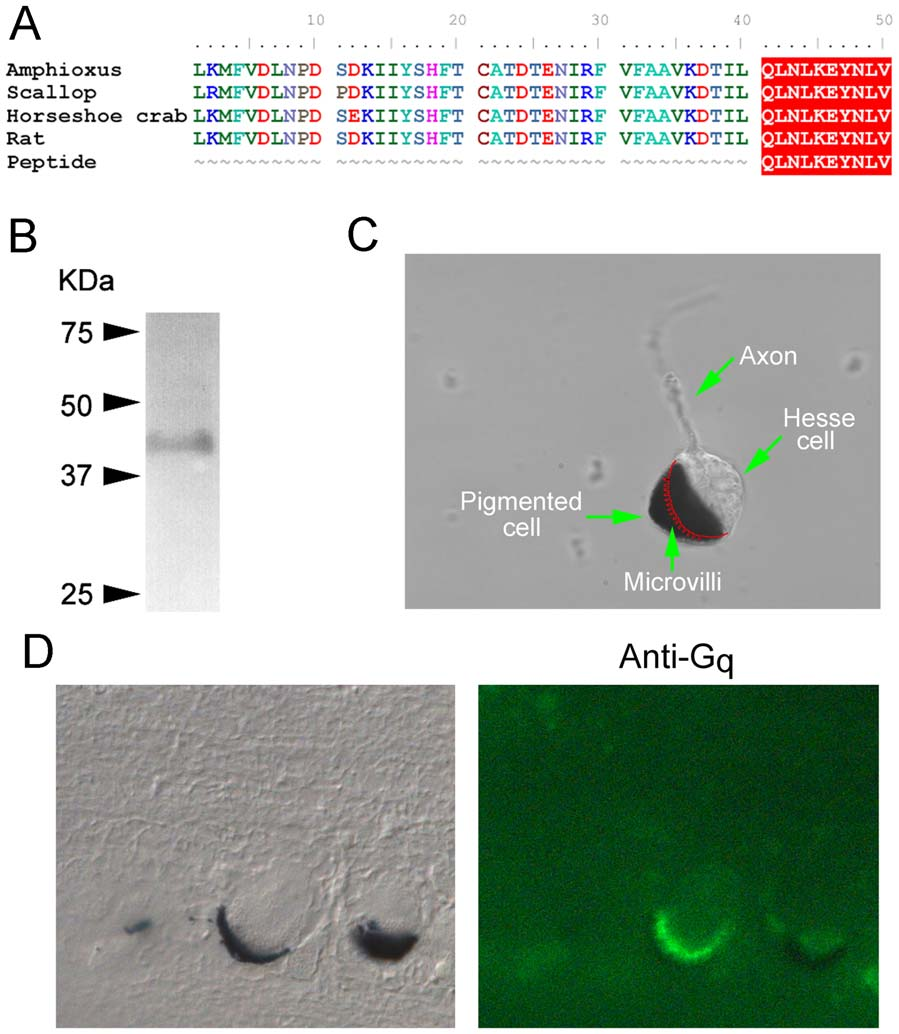
G_q_ expresses in the microvillar membrane of Hesse cells. (*A*) The sequence of the immunogenic peptide used to raise anti-G_q_ antibodies was aligned with the predicted C-terminal region of G_q_ of amphioxus, and those of other organisms. (*B*) Western blot of neural tube using anti G_q_, showing the detection of a single band of ≈42 kDa. (*C*) Morphological characteristics of the Hesse cell: the accessory pigmented cell engulfs the microvilli-covered region of the clear sensory cell; the position of the villous membrane within the occluded region is drawn in red. (*D*) *Left*: Nomarski micrograph of a 10 µm section of fixed neural tube containing two Hesse cells. *Right*: fluorescence image of the same section incubated with anti-G_q_ antibodies and Alexa Fluo 488-conjugated secondary antibodies. The immunostaining is confined to the region of the microvilli.

G_q_ has been previously identified in another species of amphioxus, *B. belcheri*, where its distribution matches the expression pattern of melanopsin [11]. We sought to obtain more detailed information on its cellular localization, to buttress the proposed role in light transduction. We found a commercial polyclonal antibody (Millipore anti-Gq/11CT) raised against a synthetic peptide that is 100% identical to the predicted C-terminal aminoacid sequence of the putative G_q_ found in the amphioxus genome. This motif is widely conserved across species, and Figure 2A shows an alignment of the immunogenic peptide sequence (QLNLKEYNLV) against the last 50 aminoacids of G_q_ of *Branchiostoma* and other organisms. In a Western blot of neural tube this antibody identified a single band (Figure 2B), with the expected molecular weight (≈42 kDa). The same antibody was then utilized in immunohistochemistry. We focused on Hesse cells, because the presence of the companion pigmented screening cell makes their identification unambiguous even in tissue sections. Figure 2C illustrates the main features of a dissociated Hesse cell: the accessory screening cell engulfs the microvilli-covered region of the sensory cell [12]; therefore, in a slice that cuts through the middle of the ocellus, the profile of the villous region (red drawing in Fig. 2C) would be revealed. The left panel of Figure 2D shows a Nomarski micrograph of a 10 µm section of fixed neural tube containing two Hesse cells; the one on the left was sliced near the middle. The right panel shows the corresponding fluorescence image, stained with anti-G_q_ antibodies and Alexa Fluo 488-conjugated secondaries. The ‘crown’ of microvilli is distinctly and selectively decorated, abutting a crescent-shaped slice of the dark accessory cell. It can be concluded that G_q_ expresses in the photo-sensitive membrane of identified Hesse cells. We sought therefore functional evidence for the participation of the G_q_-triggered cascade in light transduction.

**Figure 3:**
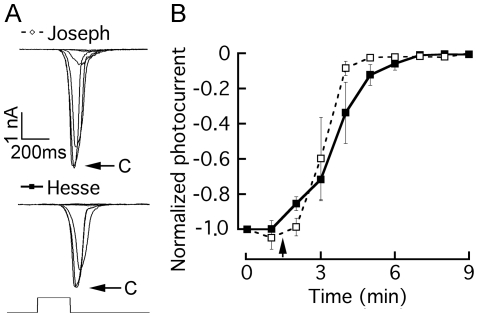
Pharmacological interference with PLC suppresses the photocurrent. The PLC antagonist U-73122 (10 µM) was applied to voltage-clamped photoreceptors stimulated every minute with a flash of light of fixed intensity. (*A*) Superimposed current traces measured in a Joseph cell and in a Hesse cell; after photocurrent stabilization, two control responses were measured (*‘c’ and arrows*), and then the drug was introduced by local superfusion, inducing a progressive decline in the peak amplitude of the light-evoked current in both cell types. (B) Time course of the effect of the PLC inhibitor, averaged for 4 Hesse cells (*solid lines/filled symbols*) and 3 Joseph cells (*dashed lines/open symbols*); error bars indicate standard error; the starting time of drug application is marked by the arrow.

Phospholipase C (PLC) is the prime target of G_q_. The compound U-73122 has been shown to inhibit the activation of PLC in a variety of experimental systems [17], [18], including microvillar invertebrate photoreceptors [19]. The effects have been reported to be irreversible. We tested this drug on the light-evoked current in voltage-clamped Joseph and Hesse cells. U-73122 was initially dissolved in DMSO at a 1 mM concentration, and an aliquot of this stock solution diluted 1∶100 in ASW to a final concentration of 10 µM. Repetitive flashes of light of sub-saturating intensity were applied every 60 sec. After attaining stability, two control responses were recorded and then the drug was rapidly introduced by local superfusion via a puffer pipette. Figure 3A illustrates the progressively decreasing photocurrents in representative cells of the two types, while Figure 3B shows a plot of the time course of changes in the peak amplitude of the light-evoked current, averaged separately for 3 Joseph cells and 4 Hesse cells. For each cell, the data were normalized with respect to the size of the first response. In both classes of cells, U-73122 caused a dramatic suppression of the photocurrent, and within ≈3–4 minutes the light response was virtually abolished. By contrast, voltage-activated currents were unaffected by the drug. Control application of ASW containing 1% DMSO produced no effect.

**Figure 4:**
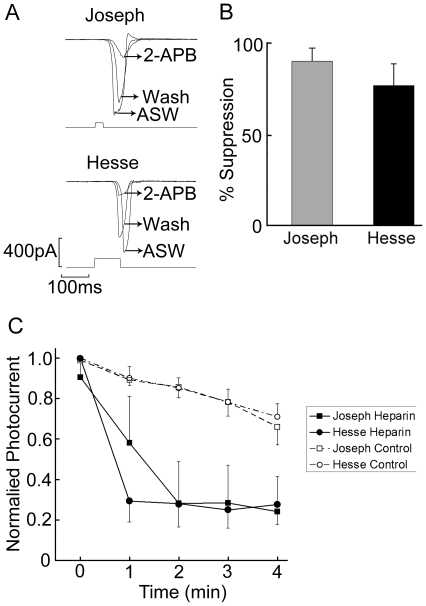
Inhibition of the light response by antagonists of the IP_3_ receptor. (*A*) Effect of 2-APB applied extracellularly at a concentration of 100 µM. Examples of photocurrent recordings in Joseph and Hesse cells before, during and after puffer application of 2-APB, highlighting the reversible inhibitory effects. Holding potential −50 mV. (*B*) Average inhibition of the light-evoked current by 2-APB; error bars indicate the standard deviation (*n = 5 for Joseph cells, n = 6 for Hesse cells*). (C) Effect of heparin. Low-molecular weight heparin was dialyzed intracellularly through the patch pipette (2 mg/ml), as voltage-clamped photoreceptors were repeatedly stimulated with a 100-ms flash of light. The peak amplitude of the photocurrent is plotted as a function of time, starting immediately after breaking the patch of membrane to access the cell interior (*Control: Joseph n = 6, Hesse n = 4; Heparin: Joseph n = 3, Hesse n = 4*).

Our previous results demonstrated that light mobilizes calcium from internal stores, and that blunting Ca elevation by chelators severely impairs the photoresponse [15]. Together with the effects of U-73122 described above, these observations likely implicate IP_3_ in the Ca-dependent regulation of the light response. To corroborate such notion, we examined the impact of antagonists of the IP_3_ receptor on the photocurrent. We first tested *2-*aminoethoxydiphenyl borate (2-APB), which has been widely utilized as an inhibitor of the IP_3_ receptor [20]. The advantages of this drug include its membrane-permeability and reversibility, thus allowing extracellular application and within-cell comparisons. The effect of 2-APB on the light response was assessed by superfusion at a concentration of 100 µM. Figure 4A shows photocurrent traces recorded in a Hesse cell and in a Joseph cell in control solution, during exposure to 2-APB, and after washing the drug. In both cases, a rapid, reversible reduction of the peak amplitude of the photocurrent occurred. Panel B of the same figure summarizes the results: the inhibition of the light response, calculated as 1-R_2-APB_/R_Control_, was 77.6%±11.6% SD for Hesse cells (n = 6), and 89.9%±7% in Joseph cells (n = 5). The somewhat lower apparent effectiveness of 2-APB in Hesse cells may hint at the greater difficulty of penetration of the drug into the microvillar region, where access is hindered by the abutting pigmented cell; however, this trend fell short of statistical significance.

Although 2-APB was initially thought to act selectively on the IP_3_ receptor, subsequent reports have questioned its alleged high specificity, showing more widespread effects on calcium-signaling, including an antagonistic action on store-operated calcium influx [21] and on SERCA pumps [22]. While 2-APB remains a useful tool for examining phosphoinositide-dependent Ca mobilization we sought additional supporting evidence for the involvement of the IP_3_R in the light response. We examined the effects of low-molecular weight heparin (<6 KDa), which has long been known to reduce the IP_3_-triggered release of calcium from the endoplasmic reticulum [23]. The effects of heparin on the light response were assessed by dialyzing it into amphioxus photoreceptors at a concentration of 2 mg/ml (dissolved in standard intracellular solution), and administering repetitive flashes of light (1/min), starting immediately after attaining the whole-cell configuration. In Figure 4C the average peak amplitude of the photocurrent, normalized by the largest response, is separately plotted as a function of time for Hesse and Joseph cells, under control conditions (standard internal solution) and with heparin dialysis. Heparin-treated photoreceptors of both types exhibited a dramatically enhanced decline of the light response over time, compared to the controls, which only show the normal rundown that typically occurs during the first few minutes of recording, before the photocurrent stabilizes. Because the outcome was similar for Hesse and Joseph cells, the values from the two cell classes were combined and subjected to an analysis of variance with repeated measurements. The difference between the control (n = 10) and heparin-treated cells (n = 7) was statistically significant (F = 29.3, df = 16, p<0.001).

**Figure 5:**
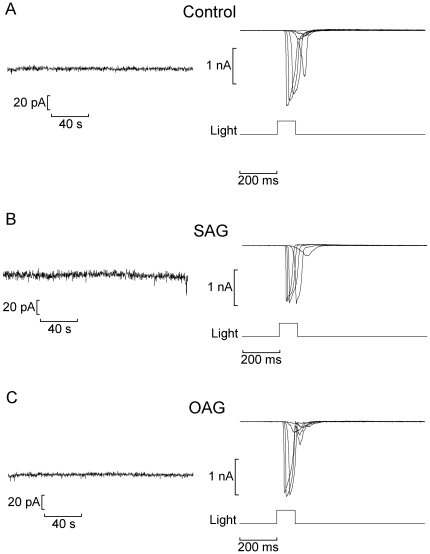
Lack of effect of diacylglycerol analogs on membrane conductance and light-evoked current. (A) Control Hesse cell dialyzed with standard internal solution. (*Left*) Recording of membrane current in the dark immediately after attaining the whole-cell configuration; (*right*) currents evoked by flashes of increasing intensity. (B,C) Similar experiments conducted in Hesse cells dialyzed with SAG (1-Stearoyl-2-arachidonoyl-*sn*-glycerol; 10 µM) and OAG (1-Oleoyl-2-acetyl-sn-glycerol; 10 µM), respectively. Neither the holding current nor the photocurrents were affected. Membrane potential was clamped at −50 mV throughout.

The above results strengthen the contention that the PLC cascade mediates light transduction in amphioxus; moreover, in conjunction with a prior demonstration of the profound inhibitory effect of intracellular BAPTA [15], they suggest that IP_3_-triggered Ca release plays a central role in this process. Hydrolysis of PIP_2_ by PLC-β generates, in addition to the soluble messenger IP_3_, membrane-bound diacylglicerol (DAG), which is also endowed with signaling properties: beyond its well-known ability to activate protein kinase C [24], DAG or its analogs and metabolites can also directly interact with ionic channels; among the reported effects are the stimulation of a cationic membrane conductance related to the light response in microvillar photoreceptors of *Lima* [25] and *Drosophila* [26], as well as the activation of heterologously-expressed TRPL channels [27]. We tested several surrogates of DAG, including the phorbol ester PMA (1 µM, n = 6), the structural analog DOG (50–100 µM, n = 4), OAG (10 µM, n = 6) and SAG (10 µM, n = 8). The compounds were either dialyzed intracellularly via the patch electrode, or applied extracellularly by rapid superfusion. The procedure entailed examining possible direct effects on membrane current, as well as alterations of the light-evoked current; to this end, I_m_ was recorded for 3 minutes in the dark as chemical stimulation was applied, followed by an assessment of photoresponsiveness by a full light-intensity series. Figure 5 illustrates the outcome obtained with two of these compounds, SAG and OAG: no change in holding current was detected, and the photocurrent showed no significant differences with respect to controls. All the DAG surrogates tested proved similarly inert in both Hesse and Joseph cells. In *Drosophila* it has been reported that poly-unsaturated fatty acids (PUFAs) such as arachidonic, linolenic and linoleic acids stimulate the light-sensitive channels [26]. Arachidonic acid can be generated from DAG by DAG lipase, and is thus able to serve as a downstream messenger in this branch of the cascade. Because the DAG analogs we utilized are not metabolized to such PUFAs, we examined the effect of direct application of arachidonic acid (5 µM; n = 4) and linolenic acid (50 µM; n = 4). Again, we failed to observe changes in membrane currents in the dark or in the light-evoked current (*not shown*).

Finally, although the support for G_q_/PLC in light signalling in amphioxus seems compelling, we examined the possible involvement of cyclic nucleotides in controlling the light response. Cyclic GMP has been proposed as an effector in the PLC-mediated light transduction of *Drosophila* [28] and *Limulus* [29], although no support has been garnered for the required cross-talk between the two pathways [30], [31]. However, the issue deserved some scrutiny, because cyclic nucleotides remain the only messenger substances for which the evidence of a *direct* effect on the gating of sensory transduction channels is undisputed, as documented in vertebrate rods [32] and in olfactory neurons [33]. We tested the effects of the poorly hydrolyzable analogs 8-Br-cGMP and 8-Br-cAMP, applied via the patch pipette (20 µM). No change in holding current was observed during intracellular perfusion in the dark while the cell vas maintained under voltage clamp at −50 mV (8-Br-cGMP: 2 Joseph and 2 Hesse cells; 8-Br-cAMP: 3 Joseph and 2 Hesse cells); subsequent tests with flashes of light showed that the photoresponse was equally unaffected (not shown).

## Discussion

The discovery of melanopsin in the vertebrate retina and the realization that it diverges from the rhodopsin of rods and cones prompted a quest for its mechanisms of action. Many of the studies addressing the nature of the biochemical cascade coupling photon absorption to ion channel gating have relied on heterologous expression, sometimes resulting in conflicting observations. In melanopsin-expressing HEK 293 cells [34] and *Xenopus* oocytes [35] it was found that, upon reconstituting a functional photopigment by exogenous retinal, a light-induced electrical response is recorded which is antagonized by inhibitors of G_q_ and PLC. By contrast, PLC was apparently not involved in the photosensitivity of Neuro-2A cells expressing melanopsin, and cyclic nucleotides seemingly played a role [36], whereas COS cell-expressed melanopsin activates transducin (G_t_) *in vitro* [37]. An important *caveat* with such an approach is that G protein-mediated cascades can be promiscuous, and implanted receptors often signal through endogenous pathways that differ from those of native cells: a case in point is mammalian rhodopsin, which in rods signals through G_t_/PDE/cGMP, but upon heterologous expression in *Xenopus* oocytes it is capable of mediating light responses by stimulating the G_q_/PLC-β/IP_3_ cascade of the host cell, which normally participates in the fertilization response [38]. It is therefore crucial to conduct functional studies utilizing *native* melanopsin-expressing cells. The progress in the physiological analysis of mammalian ipRGCs has been painstaking, as their extreme scarcity (≈1% of the retinal ganglion cell population), together with the lack of morphological landmarks to identify them, proved a formidable hurdle; the recent development of transgenic lines of animals in which such cells express a fluorescent marker [39] is a valuable approach that will help overcome such limitations. A few reports on mice ipRGCs documented phenomena that are consistent with a rhabdomeric-like light-signaling pathway: for example, the electrical response to light in ipRGCs is disrupted by peptides that preferentially antagonize G_q_, and by PLC antagonists [9]. Also, in a cone-less/rod-less background (to eliminate the potential confounding of rod and cone-driven input), a subset of retinal ganglion cells produce a light-stimulated increase in cytosolic calcium [40]; however, this Ca mobilization is only phenomenologically akin to that of rhabdomeric photoreceptors, as it appears to arise from influx (chiefly via voltage-gated Ca channels), rather than phosphoinositide-dependent release [41]. As for downstream effectors, there are suggestions based on microarray analysis that a PKC-z may be implicated [42] and immunohistochemical indications that the light-controlled conductance may be comprised of TRP-class ion channels e.g. TRP6 [43]. Many gaps, therefore, remain to be filled.

The results described in the present work strongly support the involvement of PLC signaling in the phototransduction cascade of melanopsin-expressing Joseph and Hesse cells of the neural tube of amphioxus. These observations complement the reports describing similarities between these cells and microvillar light-sensing cells of invertebrates, both in terms of morphology [12], [44], [13], [14] and electrophysiology of the photoresponse [15]. Taken together, the evidence strengthens the contention that Joseph and Hesse cells are *bona fide* members of the family of rhabdomeric photoreceptors. In addition to underscoring the central position of G_q_ and PLC for generating the melanopsin-mediated light response in amphioxus, our results provide further clues on the mechanisms implicated. A key role for PLC has recently been demonstrated in the intrinsic light sensitivity of the mammalian iris and ipRGCs [45]. In amphioxus, the robust impact of calcium and IP_3_R manipulations on light responsiveness and the lack of discernible effects by the variety of DAG analogs tested are indicative of a key role of the IP_3_ branch of the PLC cascade in the control of the photoconductance. Such a scheme has been proposed to hold for some rhabdomeric photoreceptors such as those of *Limulus* [46], whereas the importance of signaling by the lipid branch has been emphasized in other species, like *Lima* [25] and *Drosophila* [26]; in the latter, in fact, knockout of the IP_3_ receptor does not adversely affect the photoresponse [47], [48]. Establishing the soundness of such dichotomy will have to await positive confirmation of the identity of the final internal messengers that operate the light-sensitive ion channels in the various cases. Nonetheless, it is noteworthy that in murine ipRGCs, putative descendents of Hesse and Joseph cells, application of OAG reportedly also failed to alter membrane current and photoresponsiveness [9].

On the basis of molecular phylogeny, amphioxus melanopsin has been proposed to constitute a link between G_q_-coupled opsins of invertebrate and those of vertebrate circadian receptors [49], [50]. Therefore, amphioxus microvillar photo-sensitive cells provide a glimpse of the rhabdomeric lineage of light detectors in early chordate evolution, and may be regarded as ancestral to the circadian photoreceptors found in present-day vertebrates [51], [52].

## Materials and Methods

All animal procedures were conducted according to institutional guidelines.

### Cell isolation

Amphioxus (*Branchiostoma floridae*) were obtained from Gulf Specimens Marine Laboratories (Panacea, FL), and maintained in a sea-water aquarium on a diet of marine phytoplankton, and a 12/12 hr light/dark cycle. Animals were anesthetized by hypothermia, the rostral end was cut and pinned to a Sylgard-coated chamber, and the neural tube was excised. The tissue was then incubated with Pronase (Boehringer; 750 units/ml, 50 min at 22°C), followed by extensive washing in ASW supplemented with 4% fetal calf serum, and mechanical trituration with a fine-bore fire-polished Pasteur pipette. The resulting suspension was plated into a perfusion chamber mounted on the stage of an inverted microscope (Zeiss). The coverslip bottom of the chamber was pre-treated with Concanavalin-A to promote cell adhesion. Dissociated cells remain physiologically viable for several hours.

### Electrophysiological recording

Patch pipettes were fabricated from borosilicate glass, fire-polished, and filled with an intracellular solution (standard composition: 100 mM KCl, 200 K-Aspartate or K-glutamate 5 MgCl_2_, 5 Na_2_ATP, 20 NaCl, 1 EGTA, 300 Sucrose, 10 HEPES, 0.2 GTP, pH 7.3). Electrode resistance in ASW is 2–4 MΩ; series resistance was compensated electronically (maximum residual error <2 mV). Data were digitized with an analog-digital interface (Data Translation) which served also to generate stimuli, under the control of software developed in-house. Extracellular ions were exchanged by a system of reservoirs and multi-port valves, perfusing the entire flow-chamber. Alternatively, for rapid application, ‘puffer’ pipettes were lowered to a pre-set target position near the cell by a programmable positioner (Eppendorf). Pressure-ejection was controlled by solenoid-activated valves. Statistical comparisons of changes in current amplitude over time across conditions were carried out by analysis of variance with repeated measurements (program VassarStats).

### Light stimulation

Broad-band light stimuli were generated by a tungsten-halogen-quartz (THQ) light source (Oriel); IR was removed by a heat-absorbing filter (λ>800 nm). Solenoid-driven shutters (Uniblitz), calibrated neutral density filters (Melles-Griot, Zevenaar, The Netherlands), and interference filters (Omega Optical, Brattleboro, VT) controlled the duration, intensity, and wavelength of stimulation. A pin-hole restricted the illuminated region to a focused spot (≈150 µm). Alternatively, a blue LED (peak emission 470 nm) was driven by a computer-controlled precision current pump, and delivered a full-field light stimulus via a fiber optics bundle. Light was measured with a radiometer (UDT, Hawthorne, CA), and converted to effective photon flux via an in vivo calibration which made possible to compare across arrangements; the unattenuated beam intensity from the THQ illuminator was 3.53×10^15^ photons×s^−1^cm^−2^, while the maximum intensity of the LED was 2.2×10^16^ photons×s^−1^cm^−2^_._ During experimental manipulations the cells were illuminated with near-IR light (λ>780 nm; Andover Corporation long-pass filter) and viewed with the aid of a CCD camera (Sony).

### Chemicals

The phospholipase C antagonist U-73122 (1-[6-[((17β)-3-Methoxyestra-1,3,5[10]-trien-17-yl)amino]hexyl]-1H-pyrrole-2,5-dione) was from Calbiochem. Low-molecular weight heparin (<6 KDa), 2-APB (*2-*aminoethoxydiphenyl borate) and 8-Br-cAMP (8-Bromo cyclic adenosine monophosphate) were obtained from Sigma-Aldrich. PMA (phorbol ester Phorbol 12-myristate 13-acetate), DOG (1,2 Dioctanoyl-*sn*-glycerol), OAG (1-Oleoyl-2-acetyl-sn-glycerol), SAG (1-Stearoyl-2-arachidonoyl-*sn*-glycerol), arachidonic acid, and linolenic acid were purchased from Alexis biochemicals/Enzo Life Sciences. 8-Br-cGMP (8-Bromo cyclic guanosine monophosphate) was from Fluka.

### Western blots

One isolated neural tube was homogenized (teflon/glass) in the presence of protease inhibitors (100 µM PMSF, 1 µM pepstatin, and 0.1% Sigma protease inhibitor cocktail), acetone-precipitated for 1 hour at −20°C, and centrifuged 20 min at 10,000×g. The pellet was air-dried, resuspended in sample buffer and separated by SDS PAGE (8–12%). Proteins were electrotransfered (1 hour, 100 V) onto nitrocellulose membrane which was blocked overnight with 3% BSA. The membrane was then sequentially incubated with primary antibodies, washed in tris buffer saline (TBS) and incubated in alkaline phosphatase-conjugated secondary ABs. After the final washes, the nitrocellulose membrane was developed in Western Blue (Promega, Madison, WI, USA).

### Immunohistochemistry

After removal of the dorsal fin, the rostral portion of amphioxus was fixed either in 4% paraformaldehyide overnight at 4°C, or by flash-freezing in isopentane chilled in a dry-ice/liquid nitrogen slurry. Subsequently, samples were dehydrated in EtOH at −80°C for 4 days, before impregnating with polyester wax, sectioning at 10 µm and mounting onto glass slides subbed with 0.1% gelatin in 0.05% chromium potassium sulfate. Before use, the wax was dissolved by briefly dipping in 100% EtOH, and sections were re-hydrated in 70% EtOH followed by H_2_O, permeabilized in 0.2% Triton-X (5 min), and blocked with 1% goat serum (1 hr). Antibodies were applied in PBS+0.5% BSA; secondary antibodies were conjugated to Alexa Fluo- 488 (Molecular Probes), and the samples were viewed with a Zeiss Axioscope epi-fluorescence microscope.

## References

[1] Salvini-Plawen LV, Mayr E, Hecht MK, Steere WC, Wallace B (1977). The evolution of photoreceptors and eyes.. Evolut Biol.

[2] Gehring WJ (2002). The genetic control of eye development and its implications for the evolution of the various eye-types.. Int J Dev Biol.

[3] Gehring WJ, Ikeo K (1999). Pax 6: mastering eye morphogenesis and eye evolution.. Trends Genet.

[4] Arendt D, Wittbrodt J (2001). Reconstructing the eyes of urbilateria.. Phil Trans Roy Soc Lond B.

[5] Berson D, Dunn F, Takao M (2002). Phototransduction by retinal ganglion cells that set the circadian clock.. Science.

[6] Hattar S, Liao HW, Takao M, Berson DM, Yau KW (2002). Melanopsin-containing retinal ganglion cells: architecture, projections, and intrinsic photosensitivity.. Science.

[7] Provencio I, Jiang G, De Grip W, Hayes W, Rollag M (1998). Melanopsin: an opsin in melanophores, brain, and eye.. Proc Natl Acad Sci USA.

[8] Sekaran S, Lall GS, Ralphs KL, Wolstenholme AJ, Lucas RJ (2007). 2-Aminoethoxydiphenylborane is an acute inhibitor of directly photosensitive retinal ganglion cell activity in vitro and in vivo.. J Neurosci.

[9] Graham DM, Wong KY, Shapiro P, Frederick C, Pattabiraman K (2008). Melanopsin ganglion cells use a membrane associated rhabdomeric phototransduction cascade.. J Neurophysiol.

[10] Putnam NH, Butts T, Ferrier DE, Furlong RF, Hellsten U (2008). The amphioxus genome and the evolution of chordate karyotype.. Nature.

[11] Koyanagi M, Kubokawa K, Tsukamoto H, Shichida Y, Terakita A (2005). Cephalochordate Melanopsin: Evolutionary linkage between invertebrate visual cells and vertebrate photosensitive retinal ganglion cells.. Curr Biol.

[12] Eakin RM, Westfall JA (1962). Fine structure of photoreceptors in Amphioxus.. J Ultra Res.

[13] Watanabe T, Yoshida M (1986). Morphological and histochemical studies on Joseph cells of amphioxus, *Branchiostoma belcheri* Gray.. Exp Biol.

[14] Ruiz S, Anandon R (1991). Some considerations on the fine structure of rhabdomeric photoreceptors in the amphioxus, *Branchiostoma lanceolatum* (Cephalochordata).. J Hirnforsch.

[15] Gomez MP, Angueyra JM, Nasi E (2009). Light-transduction in melanopsin-expressing photoreceptors of Amphioxus.. Proc Natl Acad Sci.

[16] Fahrenkrug J, Falktoft B, Georg B, Rask L (2009). N-Linked deglycosylated melanopsin retains its responsiveness to light.. Biochemistry.

[17] Smith RJ, Sam LM, Justen JM, Bundy GL, Bala GA (1990). Receptor-coupled signal transduction in human polymorphonuclear neutrophils: effects of a novel inhibitor of phospholipase C-dependent processes on cell responsiveness.. J Pharmacol Exp Ther.

[18] Thompson AK, Mostafapur SP, Delinger LC, Bleasdale JE, Fisher SK (1991). The aminosteroid U-73122 inhibits muscarinic receptor sequestration and phosphoinositide hydrolysis in Sk-N-SH neuroblastoma cells.. J Biol Chem.

[19] Nagy K, Contzen K (1997). Inhibition of phospholipase C by U73122 blocks one component of the receptor current in Limulus photoreceptor.. Vis Neurosci.

[20] Maruyama T, Kanaji T, Nakade S, Kanno T, Mikoshiba K (1997). 2APB, 2-aminoethoxydiphenyl borate, a membrane-penetrable modulator of Ins(l,4,5)P3-induced Ca^2+^ release.. J Biochem.

[21] Gregory RB, Rychkovã G, Barritt GJ (2001). Evidence that 2-aminoethyl diphenylborate is a novel inhibitor of store operated Ca^2+^ channels in liver cells, and acts through a mechanism which does not involve inositol trisphosphate receptors.. Biochem J.

[22] Bilmen JG, Wootton LL, Godfrey RE, Smart OS, Michelangeli F (2002). Inhibition of SERCA Ca^2+^ pumps by 2-aminoethoxydiphenyl borate (2-APB): 2-APB reduces both Ca^2+^ binding and phosphoryl transfer from ATP, by interfering with the pathway leading to the Ca2+-binding sites.. Eur J Biochem.

[23] Hill TD, Berggren P-O, Boynton AL (1987). Heparin inhibits inositol trisphosphate-induced calcium release from permeabilized rat liver cells.. Biochem Biophys Res Commun.

[24] Newton AC (1997). Regulation of protein kinase C.. Curr Opin Cell Biol.

[25] Gomez MP, Nasi E (1998). Membrane current induced by protein kinase C activators in rhabdomeric photoreceptors: implications for visual excitation.. J Neurosci.

[26] Chyb S, Raghu P, Hardie RC (1999). Polyunsaturated fatty acids activate the *Drosophila* light-sensitive channels TRP and TRPL.. Nature.

[27] Estacion M, Sinkins WG, Schilling WP (2001). Regulation of *Drosophila* transient receptor potential-like (TrpL) channels by phospholipase C-dependent mechanisms.. J Physiol.

[28] Bacigalupo J, Bautista DM, Brink DL, Hetzer JF, O'Day PM (1995). Cyclic-GMP enhances light-induced excitation and induces membrane currents in Drosophila retinal photoreceptors.. J Neurosci.

[29] Johnson EC, Robinson PR, Lisman JE (1986). Cyclic GMP is involved in the excitation of invertebrate photoreceptors.. Nature.

[30] Robinson PR, Cote RH (1989). Characterization of guanylate cyclase in squid photoreceptors.. Vis Neurosci.

[31] Brown JE, Kelman ES (1996). Ca^2+^ induces an increase in cGMP-phosphodiesterase activity in squid retinal photoreceptors.. Biochem Biophys Res Commun.

[32] Fesenko EE, Kolesnikov SS, Lyubarsky AL (1985). Induction by cyclic GMP of cationic conductance in plasma membrane of retinal rod outer segment.. Nature.

[33] Nakamura T, Gold GH (1987). A cyclic nucleotide-gated conductance in olfactory receptor cilia.. Nature.

[34] Qiu X, Kumbalasiri T, Carlson SM, Wong KY, Krishna V (2005). Induction of photosensitivity by heterologous expression of melanopsin.. Nature.

[35] Panda SK, Nayak B, Campo JR, Walker JB, Hogenesch JB (2005). Illumination of the melanopsin signaling pathway.. Science.

[36] Melyan Z, Tarttelin EE, Bellingham J, Lucas RJ, Hankins MW (2005). Addition of human melanopsin renders mammalian cells photoresponsive.. Nature.

[37] Newman LA, Walker MT, Brown RL, Cronin TW, Robinson PR (2003). Melanopsin forms a functional short-wavelength photopigment.. Biochemistry.

[38] Khorana HG, Knox BE, Nasi E, Swanson R (1988). Expression of a bovine rhodopsin gene in *Xenopus* oocytes: demonstration of light-dependent ionic currents.. Proc Natl Acad Sci USA.

[39] Do MTH, Kang SH, Xue T, Zhong H, Liao HW (2009). Photon capture and signalling by melanopsin retinal ganglion cells.. Nature.

[40] Sekaran S, Russell GF, Lucas RJ, Hankins MW (2003). Calcium imaging reveals a network of intrinsically light-sensitive inner-retinal neurons.. Curr Biol.

[41] Hartwick A, Bramley JR, Yu J, Stevens KT, Allen CN (2007). Light-Evoked Calcium Responses of Isolated Melanopsin-Expressing Retinal Ganglion Cells.. J Neurosci.

[42] Peirson SN, Oster H, Jones SL, Leitges M, Hankins MW (2007). Microarray analysis and functional genomics identify novel components of melanopsin signaling.. Curr Biol.

[43] Warren EJ, Allen CN, Brown RL, Robinson DW (2006). The light-activated signaling pathway in SCN-projecting rat retinal ganglion cells.. Eu J Neurosci.

[44] Nakao T (1964). On the fine structure of the amphioxus photoreceptor.. Tohoku J Exp Med.

[45] Xue T, Do MTH, Riccio A, Jiang Z, Hsieh J (2011). Melanopsin signalling in mammalian iris and retina.. Nature.

[46] Fein A (2003). Inositol 1,4,5-trisphosphate-induced calcium release is necessary for generating the entire light response of limulus ventral photoreceptors.. J Gen Physiol.

[47] Acharya JK, Jalink K, Hardy RW, Hartenstein V, Zuker CS (1997). InsP3 receptor is essential for growth and differentiation but not for vision in *Drosophila*.. Neuron.

[48] Raghu P, Colley NJ, Webel R, James T, Hasan G (2000). Normal Phototransduction in *Drosophila* Photoreceptors Lacking an InsP3 Receptor Gene.. Mol Cell Neurosci.

[49] Koyanagi M, Terakita A (2008). Gq-coupled Rhodopsin Subfamily Composed of Invertebrate Visual Pigment and Melanopsin.. Photochem Photobiol.

[50] Terakita A, Tsukamoto H, Koyanagi M, Sugahara M, Yamashita T (2008). Expression and comparative characterization of G_q_-coupled invertebrate visual pigments and melanopsin.. J Neurochem.

[51] Plachetzki DC, Serb JM, Oakley TH (2005). New insights into the evolutionary history of photoreceptor cells.. Trends Ecol Evolut.

[52] Nasi E, Gomez MP (2009). Melanopsin-mediated light-sensing in amphioxus: a glimpse of the microvillar photoreceptor lineage within the deuterostomia.. Communicative Integrative Biol.

